# Social networking use, mental health, and quality of life of Hong Kong adolescents during the COVID-19 pandemic

**DOI:** 10.3389/fpubh.2022.1040169

**Published:** 2022-10-31

**Authors:** Lu Yu, Meng Du

**Affiliations:** Department of Applied Social Sciences, The Hong Kong Polytechnic University, Kowloon, Hong Kong SAR, China

**Keywords:** online social networking, adolescent, mental health, quality of life, social networking addiction, pandemic

## Abstract

**Background:**

During the COVID-19 pandemic, adolescents' use of social networking sites/apps has surged, and their mental health and quality of life have also been significantly affected by the pandemic and its associated social-protection measures. The present study first examined the prevalence of social networking sites/apps use and social networking addiction, the mental health status, and the health-related quality of life among Hong Kong adolescent students. We further investigated the associations of the youths' daily use of social networking sites/apps and their social networking addiction with their mental health and quality of life during the pandemic.

**Methods:**

A total of 1,147 students (age = 15.20 ± 0.53 years) recruited from 12 randomly selected local secondary schools in Hong Kong participated in a questionnaire survey in classroom settings between January and June, 2020, right after the COVID-19 outbreak. The questionnaire includes demographic characteristics and scales that measure social networking sites/apps use and social networking addiction, mental health, and quality of life.

**Results:**

Approximately 46.4% of the participants reported using social networking sites/apps often or very often, and 7.8% met the criteria for social networking addiction using Bergen's Social Media Addiction Scale. The prevalence of mild to extremely severe depression, anxiety, and stress among the adolescents stood at 39.6, 37.5, 48.8%, respectively, and the participants' physical, social, and school functioning were lower than the norms of healthy adolescents before the pandemic. Participants who used social networking sites/apps but for <3 h per day (excluding students who never used social networking sites/apps) showed significantly fewer problems of depression, anxiety, and stress than did those who spent more than 3 h per day on social networking sites/apps. Social networking addiction was found to be consistently associated with poor mental health and health-related quality of life.

**Conclusion:**

This study provides important evidence supporting the potential protective effect of guiding adolescents to use social networking sites/apps appropriately in order to mitigate their negative emotions during contexts such as that of the pandemic; it further points to the need to provide extra support to promote the well-being of young people, especially those in disadvantaged situations (e.g., non-intact family) during and after the pandemic.

## Introduction

The pandemic of COVID-19 and its variants have caused an unprecedented crisis and infected more than 640 million people worldwide (1). A series of public health measures have been implemented, including but not limited to social distancing and mandatory closure of schools (2). These measures, along with the socioeconomic impact associated with COVID-19, have presented a tremendous threat to mental health, globally, especially for young people (3). The disruption of their daily lives, challenges of online learning, limited face-to-face social interactions, increased family/parental stress, and feelings of uncertainty about the future all put children and adolescents at a high risk for mental health problems (4–6). According to the Organization for Economic Co-operation and Development, youth (aged 15–24 years) have been found to be 30–80% more likely to report symptoms of depression or anxiety and higher levels of loneliness than adults have during the pandemic (7). It is also estimated that the adverse consequences of the pandemic on youths' mental health and quality of life will be long-term, thus making continuous monitoring and prevention strategies a necessity.

Another notable issue that has emerged in the pandemic is adolescents' increased use of the Internet, and specifically their overuse of social media and social networking sites/apps (SNSs). A recent report (8) showed that there has been a significant increase in the average time people spend on social media in the United States during the pandemic. All leading social platforms reported monthly active usage growth in 2021 compared with 2019 (9). Teenagers use SNSs to keep track of news and information about the coronavirus, maintain social interactions with friends and family, and provide entertainment, such as videos (10). There are definite benefits of using SNSs during the pandemic (11, 12).

On the other hand, the use of SNSs could also be problematic. Adolescents' overuse of the SNSs increases their risk for developing social networking addiction (SNA), which is defined as the status of “being overly concerned about social networking sites (SNSs), driven by a strong motivation to log on to or use SNSs, and to devote too much time and effort to SNSs that it impairs other social activities, studies/job, interpersonal relationships, and/or psychological health and well-being” [(13), p. 175]. In a recent study in Ukraine, 26% of the student participants reported that using social media had resulted in sleep problems, which could further lead to physical and psychological problems (14). Grave concerns about overuse of SNSs during the pandemic have also been raised in the Chinese context (15). In Hong Kong, researchers had reported a high prevalence of SNA among secondary school students before the pandemic (16, 17), and the general form of Internet addiction has been growing at a staggering rate during the pandemic (18). However, there is still a lack of specific research on the amount of time that Hong Kong adolescents spend daily on SNSs and on the prevalence of social networking/media addiction (SNA) in this population, during COVID-19. Hence, the first aim of this study was to examine the general use and the excessive/problematic use of SNSs among Hong Kong adolescents during the pandemic.

Another unanswered question is what the relationships are between adolescents' use of SNSs and their mental health status, and their SNSs use and their quality of life, during COVID-19. More generally, there is a wealth of evidence, based primarily on studies done before the pandemic, supporting the notion that overuse of SNSs has a negative impact on children and adolescents' mental health (19, 20). Other studies have suggested a mutual relationship between overuse of SNSs and an increased sense of loneliness (21–23). Nonetheless, mixed findings have also been reported, suggesting that the relationship between adolescents' frequency of SNS use and their depressive/anxiety symptoms may not be significant over time (24, 25).

Still, relatively few studies have been conducted to examine the impact of SNSs use/excessive use on youths' well-being since the outbreak of the pandemic (26, 27). Among the few that have been done, Chen et al. (15) reported that school-aged children spent significantly more time on their smartphones and social media during school suspension than before, and the positive association between the children's problematic use of social media and psychological distress was stronger during the school suspension than at the baseline. On the other hand, some scholars hold positive views about using social networking sites during the pandemic. Via social networking online, adolescents can keep connected with their friends and teachers when close in-person contact is not possible, thus providing important social support that reduces the adolescents' perceived loneliness, enables them to handle negative emotions, and promotes their well-being (11, 28). In Cauberghe et al.'s study (10), online social networking was perceived by adolescents to be a constructive coping strategy to deal with their anxiety and depression during COVID-19. Abbas et al. (29) further posited that social media use provides great access for adolescents to gain health-related information, which can further lead to healthy behaviors that can prevent the spread of the virus. These findings seem to suggest that excessive social networking usage during the specific situation of COVID-19 may not necessarily be harmful to youths' mental health and quality of life, but instead may play a protective role for their well-being. Against this mixed background, the second aim of the present study was to investigate the association between Hong Kong adolescents' use and / or excessive use of SNSs during the pandemic and their mental health and their quality of life, by studying a representative sample of secondary school students in Hong Kong.

Furthermore, studies have shown that adolescents generally have displayed increased mental health problems and impairment in social functioning (30–34) since the outbreak of the pandemic. With particular reference to Hong Kong, one of the most densely populated areas in the world (35), adolescents living in this city have been facing distinct challenges in the COVID-19 pandemic. The stringent public health measures have forced people to stay at home, which on the one hand may provide opportunities for families to develop family solidarity and to support each other, but on the other hand may increase family stresses and conflicts, especially when members have to share the limited living space and resources of the home that are necessary for working and studying remotely. Indeed, studies have shown that living in small, crowded living and learning spaces with a lack of outside activity are positively associated with higher than usual levels of depression (36, 37). A dense living environment also increases the risk for community-acquired infections (38), which creates more COVID-related fear in the residents (39). At the same time, parents are experiencing high levels of stress (40, 41), which in turn affects their adolescent children's adjustment through a cascading process.

However, research specifically investigating young people's well-being in terms of both mental health and health-related quality of life in Hong Kong during the pandemic has been relatively limited (42, 43), and the findings are inconsistent. In one study, high rates of depressive symptoms (59.2%), anxiety (66%), and stress (53.7%) were reported in Hong Kong university students (44). Another study, based on a secondary school student sample, revealed that 39.2% of the participants reported being more stressed; 59.5% reported feeling greater study pressures; 24.2% were feeling more horrified; 33.2% were feeling more apprehensive, and 22% were feeling more helpless during the pandemic than they were before (43). In contrast, a longitudinal survey showed that the prevalence of suicidal ideation among secondary school students decreased from 24 to 21% after the first, large wave of COVID, with 14% having recovered from the suicidal ideation group and only 10.7% first reporting suicidal ideation in the follow-up (18). Clearly, more studies are needed in order to build a comprehensive understanding of the mental health status and quality of life of Hong Kong adolescents who are now facing a long-term disturbance following the onset of the pandemic. The third aim of the present study was to address this issue.

To summarize, the purpose of this study was threefold: (a) to examine the prevalence of Hong Kong adolescents using social networking sites (SNSs) and their incidence of problematic use (i.e., social networking addiction, SNA) during the COVID-19 pandemic, (b) to estimate the mental health status and health-related quality of life of Hong Kong adolescents during the pandemic, and (c) to investigate the relationship between different degree of SNSs use behaviors (including SNA) and adolescents' well-being under the influence of the pandemic. Specifically, we attempted to answer the following research questions:

1. How often are Hong Kong adolescents using SNSs, and what is the percentage of adolescents showing social networking addiction (SNA), during the pandemic?
2. What is the mental health status of Hong Kong adolescents during the pandemic? What is their quality of life?
3. What is the relationship between different degrees of SNS use, including SNA, and Hong Kong adolescents' mental health? What is the relationship between different degrees of their SNSs use and their health-related quality of life?

## Methods

### Participants and procedure

This study was part of a larger longitudinal project investigating the positive youth development and Internet use of Hong Kong adolescents. The target population was Hong Kong junior secondary school students. Based on a two-stage cluster sampling, we first randomly selected 20 schools from all 471 Hong Kong local secondary schools; then all students studying in Secondary One (i.e., Grade 7) in the selected schools were invited to join the survey. The survey was conducted between January and June, 2020, right after the COVID-19 outbreak. Students who agreed to participate in the study completed the questionnaire in classroom settings. A trained research staff member administered the questionnaire survey without the presence of any school staff. Strict confidentiality was maintained during the data collection. Participants' completed questionnaires were kept strictly confidential.

### Ethical considerations

The project was approved by the Human Subjects Ethics Sub-Committee (HSESC) of the first author's institution (Reference No.: HSEARS20180326015). After obtaining the approval from the participating schools, students were invited to participate in the study on a voluntary basis. Both parents' and students' written informed consents were obtained before the data collection. In order to ensure anonymity and confidentiality, participants were not identified individually and data were treated in aggregate manners for overall analyses. Data collected from the questionnaires were handled by members of the Research Team only, and the records were securely stored such that only members of the Research Team were able to gain access to.

### Instruments

Demographic information on the participants was collected with a list of items that were drafted by the research team and had been used in previous large-scale studies (45, 46). Students' social networking addiction (SNA), use of SNSs, mental health, and quality of life were measured by validated Chinese versions of questionnaires, as described below.

#### Bergen social media addiction scale

The validated Chinese six-item version of the Bergen Social Media Addiction Scale [BSMAS; (47)] was adopted to assess the participants' addiction to social networking (48). The BSMAS measures six core symptoms of behavioral addiction (i.e., salience, mood modification, tolerance, withdrawal, conflict, and relapse), which were based on Griffith's (49) model. Participants responded to each item using a five-point rating scale to indicate the frequency of the described behavior they had displayed in the past year (1= very rarely; 2 = rarely; 3 = sometimes; 4 = often; 5 = very often). Higher scores on the BSMAS indicate greater problematic use of social media or SNSs. In the present study, we adopted the criterion (50) that participants with a total scale score above 19 would be identified as being at high risk for SNA. The Cronbach's alpha of the BSMAS in the present study was 0.89.

Two additional questions were asked to assess the adolescents' self-perceived daily amount of time spent on SNSs and their frequency of usage. The first question asked the respondents “how much time have you spent on SNSs daily in the past month,” and they answered using a five-point rating scale (1 = never; 2 = < 1 h; 3 = 1–2 h; 4 = 2–3 h; 5 = more than 3 h). Second, the respondents answered the question “how frequently did you use SNSs in the past week” on a five-point rating scale (1 = never; 2 = occasionally; 3 = sometimes; 4 = often; 5 = very often).

#### Depression anxiety stress scale-21

The students' mental health was measured by the Chinese version of the Depression Anxiety Stress Scale-21 [DASS 21, (51)], which has been a widely adopted measure of emotional states of depression (seven items), anxiety (seven items), and stress (seven items). Participants indicated how strongly each statement applied to them over the previous week using a four-point rating scale (0 = did not apply to me at all; 1 = applied to me to some degree or some of the time; 2 = applied to me to a considerable degree or a good part of time; 3 = applied to me very much or most of the time). Higher scores represented a higher level of emotional problems. The Cronbach's alpha for the three subscales in this study were 0.84 (depression), 0.91 (anxiety), and 0.86 (stress).

#### Pediatric quality of life inventory

The Pediatric Quality of Life Inventory [PedsQL, (52)] is a well-validated brief measure of health-related quality of life in children and adolescents and has been validated in Chinese population (53, 54). It is a self-reported questionnaire for adolescents that has four dimensions: physical functioning (eight items), emotional functioning (five items), social functioning (five items), and school functioning (five items). For each item, the participants rated the frequency at which the described phenomena occurred in their life during the month prior to the survey, using a five-point response scale (0 = never a problem; 1 = almost never a problem; 2 = sometimes a problem; 3 = often a problem; 4 = almost always a problem). We did not adopt the five items of the emotional functioning subscale in the present study because the DASS21 had already measured the participants' emotional status. The PedsQL is reverse-coded and computed into standardized scores (54), with higher scores representing a better health-related quality of life. Based on the present sample, the Cronbach's alpha coefficients for this study were 0.87 for physical functioning, 0.89 for social functioning, and 0.78 for school functioning.

### Data analysis

We used SPSS 26.0 statistical software for data analysis in the present study. For the first two research questions, descriptive statistics were first computed to provide a profile of the prevalence of the use of SNSs and the incidence of SNA among the Hong Kong adolescents during the pandemic, as well as their mental health status and health-related quality of life. To examine the association between the amount of time that the adolescents used SNSs and their mental health and also their health-related quality of life, two MANOVA were performed, controlling for age and gender: in the first MANOVA, depression, anxiety, and stress were treated as dependent variables (DV) and the participants' self-reported daily amount of time using SNSs was treated as an independent variable (IV); in the second MANOVA, physical, social, and school functioning served as the DVs with the same IV. To investigate the relationships between SNA and the adolescents' mental health and quality of life statuses, six multiple regression analyses were performed. In each regression model, one indicator of the participants' mental health (i.e., depression, anxiety, or stress) or health-related quality of life served as the DV; the demographic characteristics of gender, age, family intactness, and immigration status (whether the individual had been born in Hong Kong or elsewhere) were entered into the first block, and their SNA scores (indicated by the participants' scores on the BSMAS) was entered into the second block.

## Results

### Demographic characteristics of the participants

The participants comprised 1,147 students (602 females and 546 males; 21 did not report their gender) who were recruited from 12 secondary in Hong Kong. The mean age of the participants was 15.20 years (SD = 0.53 years) at the time of data collection. The majority of the students were born in Hong Kong (*N* = 1024; 87.6%) and were growing up in intact families (*N* = 868; 74.3%).

### Prevalence of SNSs use and of SNA

First, in terms of the amount of time that the participants spent on SNSs, 30.8% of the students spent 2–3 h per day, while 28.2% spent more than 3 h; and 46.4% of the participants reported that they used SNSs often or very often. Second, different problematic use-of-SNS behaviors were reported by the students and are summarized in Table 1. The students' mean total BSMAS score was 13.04 ± 4.96. Using the cutoff score of 19 (50), 7.8% of the students could be identified as having SNA. Among the six SNA behaviors, “spend a lot of time thinking about social media or planning how to use it” was the most prevalent one, with 15.8% of the participants reporting that they were preoccupied with SNSs (salience) often or very often. Notably, 10.3% of the students expressed that they “use[d] social media so much that it has had a negative impact on your job/studies” (conflict), and 9.9% reported that they often or very often “use[d] social media in order to forget about personal problems” (mood modification).

**Table 1:** Descriptive statistics of the participants' SNSs use and SNA behaviors.

**Variable**	**Mean**	**SD**	**Never**	**Occasionally**	**Sometimes**	**Often**	**Very often**
SNSs use: frequency	3.35	0.95	28 (2.4%)	196 (16.9%)	399 (34.3%)	423 (36.4%)	116 (10.0%)
	**Mean**	**SD**	**Never**	<**1 h**	**1–2 h**	**2–3 h**	≥**3 h**
SNSs daily use: amount of time	3.73	1.31	256 (22.1%)	218 (18.8%)	357 (30.8%)	209 (18.0%)	118 (10.2%)
**BSMAS items**	**Mean**	**SD**	**Very rarely**	**Rarely**	**Sometimes**	**Often**	**Very often**
1. You spend a lot of time thinking about social media or planning how to use it.	2.57	1.03	205 (17.6%)	316 (27.1%)	459 (39.4%)	139 (11.9%)	45 (3.9%)
2. You feel an urge to use social media more and more.	2.18	0.98	320 (27.5%)	430 (37.0%)	321 (27.6%)	62 (5.3%)	29 (2.5%)
3. You use social media in order to forget about personal problems.	2.17	1.06	372 (32.0%)	374 (32.2%)	302 (26.0%)	75 (6.5%)	39 (3.4%)
4. You have tried to cut down on the use of social media without success.	2.03	1.03	430 (37.1%)	395 (34.1%)	235 (20.3%)	67 (5.8%)	32 (2.8%)
5. You become restless or troubled if you are prohibited from using social media.	1.92	1.00	491 (42.3%)	371 (31.9%)	229 (19.7%)	39 (3.4%)	32 (2.8%)
6. You use social media so much that it has had a negative impact on your job/studies.	2.19	1.06	361 (31.1%)	384 (33.1%)	297 (25.6%)	75 (6.5%)	44 (3.8%)

### Mental health and health-related quality of life

Table 2 shows the means and standard deviations of the participants' scores on the DASS21. Their scores for the depression, anxiety, and stress subscales were doubled to calculate a final score for the identification of different levels of negative emotions. According to the recommended cutoffs (53), participants were categorized into groups of different levels of negative emotions. It was revealed that the prevalence of mild to extremely severe depression in the participants stood at 39.6%, anxiety was at 37.5%, and stress was at 48.8%. In particular, 14.4% of the students reported severe or extremely severe depression, 9.5% reported severe or extremely severe anxiety, and 14.8% had severe or extremely severe stress. The students' standardized scores on the physical, social, and school functioning subscales of the PedsQL were 78.80 ± 17.65, 80.53 ± 18.82, and 75.85 ± 18.11, respectively. These scores were significantly lower than the norm scores of healthy children and adolescents in different Chinese contexts before the pandemic (55).

**Table 2:** Descriptive statistics of the participants' levels of depression, anxiety, and stress.

**Variable**	**Mean**	**SD**	**Mild**	**Moderate**	**Severe**	**Extremely severe**	**Severe or extremely severe**	**Mild to extremely severe**
Depression	4.66	4.90	115 (10.7%)	158 (14.8%)	60 (5.6%)	91 (8.5%)	151 (14.4%)	424 (39.6%)
Anxiety	4.30	4.22	123 (11.5%)	176 (16.5%)	46 (4.3%)	56 (5.2%)	102 (9.5%)	401 (37.5%)
Stress	5.19	4.64	144 (13.0%)	233 (21.0%)	93 (8.4%)	71 (6.4%)	164 (14.8%)	541 (48.8%)

### Relationship between SNS use and SNA and the adolescents' well-being

Results of two MANOVA are summarized in Table 3. The students' daily amount of time spent on SNSs was significantly associated with their mental health (*F* = 3.24, Wilk's lambda = 0.96, *p* < 0.001, η^2^*p* = 0.01, 90% CI 0.00–0.02) and their health-related quality of life (*F* = 2.41, Wilk's lambda = 0.97, *p* < 0.01, η^2^*p* = 0.01, 90% CI 0.00–0.02). *Post-hoc* comparisons further revealed that students who spent “ < 1 h,” “1–2 h,” and “2–3 h” per day on SNSs had lower scores on the scales for depression, anxiety, and stress than students who spent “≥ 3 h” per day did, but interestingly, there was no significant difference between the group who never used SNSs and the group who used SNSs for more than 3 h per day. In terms of health-related quality of life, *post-hoc* multiple comparisons showed that only in school functioning did the group who never used SNSs score higher than the group who reported their daily SNSs usage was 3 h or more. No other significant differences among the groups were observed.

**Table 3:** Results of MANOVA comparing the mental health and quality of life of the participants in conjunction with their daily amount of SNSs.

**Indicators**	**G1: never**	**G2: < 1 h**	**G3: 1–2 h**	**G4: 2–3 h**	**G5: ≥3 h**	**MANOVA**		
	**M (SD)**	**M (SD)**	**M (SD)**	**M (SD)**	**M (SD)**	***F***	**η^2^p**	**Multiple comparisons**
Quality of life						2.41**	0.01	
PhyFun	84.67 (25.89)	85.24 (15.81)	86.25 (14.93)	85.21 (14.33)	82.75 (16.87)			
SocFun	87.41 (19.63)	78.92 (20.46)	80.21 (17.68)	81.57 (18.21)	79.91 (19.02)			
SchFun	85.00 (20.57)	75.50 (18.37)	77.54 (17.33)	76.50 (17.01)	73.45 (19.10)			G1>G5
Mental health						3.24***	0.01	
Depression	4.56 (6.27)	4.12 (4.85)	4.28 (4.89)	3.91 (4.19)	5.87 (5.20)			G2&G3&G4 < G5
Anxiety	4.16 (6.01)	3.63 (3.72)	4.03 (3.98)	3.86 (3.96)	5.37 (4.65)			G2&G3&G4 < G5
Stress	4.44 (5.52)	4.54 (4.42)	4.84 (4.38)	4.70 (4.25)	6.42 (4.93)			G2&G3&G4 < G5

SNSs use, the amount of time the participants spent daily on the internet during the past month; PhyFun, Physical functioning; SocFun, Social functioning; SchFun, School functioning.

** p < 0.01,

*** p < 0.001.

Multiple regression analyses were performed to examine the relationships between SNA and the students' mental health and their quality of life. For the regression on mental health indicators (Table 4), family non-intactness was positively associated with the students' levels of depression (β = 0.08; *p* < 0.01), anxiety (β = 0.08; *p* < 0.01), and stress (β = 0.07; *p* < 0.05), suggesting that students from non-intact families had more symptoms of depression, anxiety, and stress than their peers from intact families did. After controlling for the demographic variables, the students' scores on the BSMAS scale–that is, their SNA scores–were positively associated with depression (β = 0.25; *p* < 0.001), anxiety (β = 0.28; *p* < 0.001), and stress (β = 0.29; *p* < 0.001). With regard to their health-related quality of life (Table 5), the regression results showed that the students' SNA scores were negatively associated with their physical functioning (β = −0.21, *p* < 0.001), social functioning (β = −0.17, *p* < 0.001), and school functioning (β = −0.23, *p* < 0.001).

**Table 4:** Results of multiple regressions on the predictive effects of SNA on the participants' mental health indicators.

	**DEP**					**ANX**					**STR**				
**Model**	***B***	**SE B**	**β**	***R* ^2^**	**_Δ_ *R* ^2^**	***B***	**SE B**	**β**	***R* ^2^**	**_Δ_ *R* ^2^**	***B***	**SE B**	**β**	***R* ^2^**	**_Δ_ *R* ^2^**
Block 1				0.01	0.01**				0.02	0.02***				0.02	0.02***
Gender	−0.22	0.30	−0.02			−0.60	0.25	−0.07**			−0.49	0.27	−0.05		
Age	0.26	0.31	0.03			0.05	0.26	0.01			0.42	0.28	0.05		
Born in HK	0.59	0.52	0.04			−0.15	0.44	−0.01			0.34	0.48	0.02		
Intact family	0.96	0.36	0.08**			0.78	0.31	0.08**			0.72	0.32	0.07*		
Block 2				0.08	0.06***				0.10	0.08***				0.10	0.08***
SNA	0.25	0.03	0.25***			0.24	0.03	0.28***			0.27	0.03	0.29***		

DEP, depression; ANX, anxiety; STR, stress.

Gender: 0, female; 1, male.

Immigration status: 1, born in Hong Kong; 0, not born in Hong Kong.

Family intactness: 0, intact family; 1, non-intact family; SNA, participants' scores on the BSMAS.

* p < 0.5,

** p < 0.01,

*** p < 0.001.

**Table 5:** Results of multiple regressions on the predictive effects of SNA on the participants' health-related quality of life indicators.

	**PHY**					**SOC**					**SCH**				
**Model**	***B***	**SE B**	**β**	***R* ^2^**	**_Δ_ *R* ^2^**	***B***	**SE B**	**β**	***R* ^2^**	**_Δ_ *R* ^2^**	***B***	**SE B**	**β**	***R* ^2^**	**_Δ_ *R* ^2^**
Block 1				0.01	0.01				0.00	0.00				0.01	0.01**
Gender	1.21	0.96	0.04			−1.75	1.14	−0.05			−1.19	1.08	−0.03		
Age	−0.52	0.98	−0.02			0.35	1.16	0.01			−1.33	1.10	−0.04		
Born in HK	−0.90	1.67	−0.02			0.10	1.97	0.00			−1.16	1.86	−0.02		
Intact family	−1.73	1.11	−0.05			−1.03	1.32	−0.02			−3.78	1.25	−0.09**		
Block 2				0.05	0.04***				0.03	0.03***				0.06	0.05***
SNA	−0.67	0.10	−0.21***			−0.66	0.12	−0.17***			−0.84	0.11	−0.23***		

PHY, physical functioning; SOC, social functioning; SCH, functioning at school.

Gender: 0, female; 1, male.

Immigration status: 1, born in Hong Kong; 0, not born in Hong Kong.

Family intactness: 0, intact family; 1, non-intact family; SNA, participants' scores on the BSMAS.

** p < 0.01,

*** p < 0.001.

## Discussion

The present study investigated Hong Kong adolescents' use of social networking sites and their social networking addiction (SNA); their mental health and health-related quality of life status; and the associations of their SNSs use and social networking addiction with their well-being during the COVID-19 pandemic. We found that nearly half of the participants reported using SNSs often or very often, while the prevalence of SNA was no higher than the reported rates before the pandemic (56). The students' mental health status and their health-related quality of life were at a low level compared with findings for those attributes before the pandemic (55). Different amounts of time spent using SNSs daily were associated differentially with negative emotions, while SNA was consistently associated with poor mental health status and poor health-related quality of life. This study's findings point to a potential protective effect on youths' mental health from using SNSs appropriately during the pandemic.

Frequent use of SNSs by the adolescents during the pandemic was found to be a common phenomenon, and the amount of time they spent on SNSs was generally associated with their well-being. Roughly one-third of the participants (30.8%) reported that they spent ~2–3 h daily on SNSs, and 28.2% reported using SNSs for more than 3 h per day. Only <20% of the students reported that they never or only occasionally used SNSs. When we further compared mental health and health-related quality of life indicators among the students with their different usage times for SNSs, the results showed that the students who spent from <1 h to ~2–3 h per day using SNSs reported lower levels of depression, anxiety, and stress than did the students who spent more than 3 h on SNSs per day, while the group of students who never used SNSs had a level of negative emotions that was comparable with that of the group with more than 3 h daily on SNSs. Contrary to other recent findings showing that the increase in the time spent using SNSs was associated with anxiety problems during the pandemic [see the meta-analysis by Lee et al. (57)], our findings suggest that using SNSs for 3 h per day or somewhat less can have beneficial effects on adolescents' mental health during the pandemic. The SNSs can provide a convenient and safe channel for youths to use for communicating and connecting with their peers, families, and teachers when face-to-face interaction is restricted; SNSs also give adolescents access to the most updated information with reference to their studies, life, health, and the pandemic. Our present findings support the positive effect of SNSs usage and offer preliminary evidence on how much time spent on SNSs may be appropriate for adolescents without compromising their physical and mental health.

Nonetheless, we also found that addiction to social networking remained associated with increased depression, anxiety, and stress symptoms and lower scores for physical, social, and school functioning. Although this finding is consistent with those of existing studies supporting the harmful effects of SNA on adolescents' health (16, 17), our results further indicate that even during the pandemic, with online interaction having become a new normal, excessive and problematic use of the SNSs can undermine rather than promote adolescents' well-being. Excessive use of SNSs cannot help adolescents address their emotional problems, reduce their sense of loneliness, or promote their deteriorated social and school functioning associated with the pandemic. Instead, uncontrollable use of SNSs has been found to lead one to neglect other beneficial aspects of one's life during the pandemic (e.g., academic work, physical exercise, offline interpersonal communication), and that can further result in negative emotions and impaired functioning (58). In particular, people with SNA are described as “experiencing a constant urge to check their social networks for new information and updates because of the fear of missing out” [(59), p. 6]. Under the unique circumstances of COVID-19, such an urge may become stronger, thus causing even more anxiety and stress.

It should be noted that such associations between SNA and mental health problems, as well as low quality of life, could be a reverse causation–that is, adolescents' existing mental health issues could result in their problematic use of the SNSs. Prior studies have found that young people who are suffering from different health and mental health problems tend to develop a pattern of using SNSs as a coping strategy to decrease their feelings of loneliness, uncertainty, and powerlessness, to gain comfort and support from others, and to maintain a sense of belongingness to their peer group (60–64). There has also been evidence for a bi-directional relationship between SNA and mental health problems: people with existing psychological problems are more likely to indulge in SNSs use to avoid or reduce their negative emotions, while compulsive use of SNSs generates additional stress and damages their well-being (64, 65). The pandemic and its associated preventive measures have jeopardized adolescents' quality of life and mental health, and that may further increase the risks that the adolescents will develop SNA. To clarify this relationship, future studies should adopt a longitudinal design that involves multiple waves of data collection of not only SNA and well-being indicators, but also of potential mediators [e.g., satisfaction of needs (66); emotional competence (67); and self-control (68)] and of moderators [e.g., offline social capital (69); mindfulness (70)]; and specific contextual factors, such as the pandemic-related social policies.

With reference to adolescents' well-being during the pandemic, 39.6% of the Hong Kong secondary school students in the present study experienced mild to extremely severe levels of depression, 37.5% experienced mild to extremely severe levels of anxiety, and 48.8% experienced mild to extremely severe levels of stress. The occurrence rates of these negative emotions were significantly higher than the rates reported for the same emotions before the pandemic (55). Participants also had significantly lower scores for their levels of physical functioning, social functioning, and school functioning as measured by the PedsQL than the norms for healthy teens (71–73). These findings further support the notion that the pandemic has been detrimental to young people's mental health and quality of life, worldwide (74–77). A recent study of university students in Hong Kong (78) identified “living alone” and “[having] experienced economic disadvantage” as risk factors for university students' negative emotional states during the pandemic. In our study, we also found that students from non-intact families (e.g., a divorced-parents family or a single-parent family) reported higher levels of depression, anxiety, and stress than their peers living in intact families did. In the face of the enduring pandemic, promoting the well-being of the general youth population should be given an especially high priority in terms of policy, research, and services, with special attention and extra resources provided to young people in disadvantaged situations, such as those with economic difficulties and insufficient family support, because they are more severely and disproportionately affected by the pandemic.

Adolescents' online social networking behaviors and their problematic use of SNSs have drawn considerable research attention in the past few years (56). However, the relationships between the daily amount of time adolescents spend on SNSs and their well-being during the pandemic have been inconclusive. Using a representative sample of Hong Kong secondary school students, the present study provides meaningful theoretical and practical implications by differentiating the associations between different dosages of SNS use and adolescents' well-being, and by verifying the negative relationship between youths' social networking addiction and their mental health as well as their health-related quality of life, especially under the influence of the pandemic. In terms of theory, the present findings provide insights into the positive side of SNSs use by youths and their improved well-being, and thus contribute to the current literature, which has mainly highlighted the negative effects of SNSs usage on young people's mental health (79–81). Especially under the particular circumstances of the COVID-19 pandemic, appropriate use of SNSs can help young people to maintain their mental health through fulfilling their need to be socially connected and their desire to follow pandemic-related updates (82). Our findings also add to the existing knowledge by identifying an ideal range for the daily amount of time (from somewhat <1–3 h) that an adolescent can spend on SNSs without causing evident adverse effects on their well-being.

In practical terms, our findings corroborate prior findings suggesting that both excessive use of SNSs (more than 3 h per day), and SNA, are likely to jeopardize youths' mental health and well-being, and that the pandemic has disproportionately affected youths that are in disadvantaged situations (e.g., non-intact families). First, while SNSs have increasingly become a necessary and important part of young people's lives, there is a critical need to develop evidence-based effective strategies to help adolescents harness their use of SNSs to the benefit of their well-being. Second, policy makers at different levels and also school teachers need to pay extra attention to disadvantaged families and their children, in an effort to tackle the unique challenges faced by this population. Third, recommendations on the amount of daily time devoted to using SNSs should be made and incorporated into the existing guidelines for youths' healthy use of the Internet.

In interpreting the findings of the present study, several limitations should be considered. First, the data were collected from self-reported questionnaires, which makes the results likely to have been affected by responses with social desirability bias. However, the anonymous nature of the survey, the non-presence of classroom teachers in the questionnaire administration process, and the reassurance of the data confidentiality potentially reduced the social desirability bias. Still, it would be ideal to also collect data from parents, teachers, or other informants on the amount of time that adolescents really spend on SNSs daily, and on their school, social, and physical functioning. New technology that simply records the exact amount of time that a person uses SNSs per day could be further incorporated into such studies in the future. Second, this study's cross-sectional data cannot rule out the possibility of reverse or recursively causal relationships, so longitudinal data are warranted. Third, we asked the respondents to report only their time spent on SNSs, and did not inquire how they used the SNSs, nor did we ask the purposes of the usage, all of which could play an equal, if not more important, role in clarifying the relationships between SNSs use and youths' well-being. In future studies we must include measures of adolescents' different types of SNSs use, to enhance our understanding of the mechanisms underlying these relationships. Despite these limitations, however, the present study contributes to our understanding of the relationships between Hong Kong adolescents' SNSs usage and their mental health status and health-related quality of life, and also the relationships between their SNSs usage and their well-being during the unique context of the COVID-19 pandemic.

## Conclusion

Based on a representative sample of Hong Kong secondary school students, the present study examined Hong Kong adolescents' use of social networking sites (SNSs), social networking addiction (SNA), mental health, and health-related quality of life status as well as their relationships during the pandemic. Frequent use of SNSs was common among Hong Kong adolescents but the prevalence rate of SNA was not increased; students' mental health status and their health-related quality of life were at a low level. We found that using SNSs for 3 h per day or somewhat less can have beneficial effects on adolescents' mental health, while excessive and problematic use of the SNSs can undermine rather than promote adolescents' well-being even during the pandemic. The findings of the present study can guide the efficient mental health promotion strategy for adolescents under the influence of the pandemic and provide recommendations for youths' healthy use of the Internet.

## Data availability statement

The raw data supporting the conclusions of this article will be made available by the authors, without undue reservation.

## Ethics statement

The project was approved by the Human Subjects Ethics Sub-Committee (HSESC) of The Hong Kong Polytechnic University (Reference No.: HSEARS20180326015). Written informed consent to participate in this study was provided by the participants' legal guardian/next of kin.

## Author contributions

LY conceptualized and designed the study, collected the data, interpreted the data, drafted the manuscript, and approved the final manuscript as submitted. MD collected the data, interpreted the data, drafted the manuscript, and approved the final manuscript as submitted. All authors contributed to the article and approved the submitted version.

## Funding

The work described in this manuscript was fully supported by a grant from the Research Grants Council of the Hong Kong Special Administrative Region, China (Project No. PolyU 15604618). The preparation and publication of the work was funded by the Departmental Large Project Funding Scheme, Department of Applied Social Science, The Hong Kong Polytechnic University (Project No. P0041161).

## Conflict of interest

The authors declare that the research was conducted in the absence of any commercial or financial relationships that could be construed as a potential conflict of interest.

## Publisher's note

All claims expressed in this article are solely those of the authors and do not necessarily represent those of their affiliated organizations, or those of the publisher, the editors and the reviewers. Any product that may be evaluated in this article, or claim that may be made by its manufacturer, is not guaranteed or endorsed by the publisher.
